# Protective role of N-acetylcysteine (NAC) on human sperm exposed to etoposide

**DOI:** 10.1186/s12610-018-0082-2

**Published:** 2019-02-07

**Authors:** João Baetas, Ana Rabaça, Ana Gonçalves, Alberto Barros, Mário Sousa, Rosália Sá

**Affiliations:** 1 Laboratory of Cell Biology, Department of Microscopy, Institute of Biomedical Sciences Abel Salazar (ICBAS), University of Porto (UP), Rua Jorge Viterbo Ferreira, 228, 4050-313 Porto, Portugal; 2 Faculty of Sciences, University of Porto, 4169-007 Porto, Portugal; 3 Centre for Reproductive Genetics A. Barros (CGR), Av. do Bessa, 240, 1° Dto. Frente, 4100-012 Porto, Portugal; 4 Department of Genetics, Faculty of Medicine, University of Porto, Alameda Prof. Hernâni Monteiro, 4200-319 Porto, Portugal; 5 Health Institute of Research and Innovation (IPATIMUP/i3S), University of Porto, Rua Alfredo Allen, 208, 4200-135 Porto, Portugal; 6 Multidisciplinary Unit for Biomedical Research (UMIB), University of Porto, Rua Jorge Viterbo Ferreira, 228, 4050-313 Porto, Portugal

**Keywords:** N-acetylcysteine (NAC), Etoposide, Sperm DNA fragmentation, Sperm oxidative stress

## Abstract

**Background:**

Although recent progress in cancer treatment has increased patient survival and improved quality of life, reproductive side effects are still for concern. One way to decrease gonadal impairment is to use cytoprotectors. In testicular cancer, etoposide is generally used in combination with other agents, but there are no in-vitro studies of sperm exposure to etoposide and cytoprotectors, namely N-acetylcysteine (NAC).

**Methods:**

Twenty semen samples were individually divided into five groups: control, incubation with NAC alone, incubation with etoposide alone, sequential exposure of NAC followed by etoposide (pre-treatment) and sequential exposure of etoposide followed by NAC (post-treatment). Sperm characteristics, chromatin condensation (aniline blue), DNA fragmentation (TUNEL), oxidative stress (OxyDNA labelling) and glutathione quantification were used to evaluate the capabilities of NAC as a prophylactic (pre-treatment) or ameliorator (post-treatment) agent over the effects caused in sperm during in-vitro exposure to etoposide.

**Results:**

No deleterious effects were observed on sperm motility or sperm membrane integrity. Results revealed that prophylactic use of NAC (pre-treatment) increased rates of immature sperm chromatin as compared to ameliorator use of NAC (post-treatment), and increased rates of sperm DNA fragmentation in relation to controls. Pre and post-treatment with NAC increased oxidative levels in comparison to controls, but also increased levels of cellular antioxidant glutathione.

**Conclusions:**

The results indicate that NAC has the ability to counteract etoposide-induced toxicity rather than preventing the etoposide cytotoxic effects over sperm DNA, suggesting that the administration of NAC to cells formerly exposed to etoposide is preferable to its prophylactic use. As the results evidenced that NAC seems to be more efficient in attenuating sperm etoposide cytotoxic effects instead of being used as a chemoprophylactic agent, this reinforces the idea that there might be a new NAC mechanism over DNA.

## Résumé

**Contexte:**

Bien que les récents progrès dans le traitement des cancers aient augmenté la survie des patients et amélioré leur qualité de vie, les effets secondaires sur la reproduction restent encore des motifs d’inquiétude. Une façon de réduire les altérations gonadiques consiste en l’utilisation de cytoprotecteurs. Dans le cancer du testicule, l’étoposide est habituellement utilisée en association avec d’autres agents, mais il n’existe aucune étude in vitro de l’exposition des spermatozoïdes à l’étoposide et à des cytoprotecteurs, notamment la N-acétylcystéine (NAC).

**Matériel et Méthodes:**

Vingt échantillons de sperme ont été répartis en cinq groupes : témoins, incubés avec NAC seule, incubés avec étoposide seul, exposés séquentiellement à NAC puis à étoposide (pré traitement), et exposés séquentiellement à étoposide puis à NAC (post traitement). Les paramètres spermatiques, la condensation de la chromatine (bleu d’aniline), la fragmentation de l’ADN (TUNEL), le stress oxydatif (marquage OxyDNA) et la quantification du glutathion ont été utilisés pour évaluer les capacités de NAC comme agent prophylactique (pré traitement) ou comme améliorateur (post traitement) des effets causés sur les spermatozoïdes lors d’une exposition in vitro à l’étoposide.

**Résultats:**

Aucun effet délétère n’a été observé sur la mobilité ou sur l’intégrité de la membrane des spermatozoïdes. Les résultats montrent que l’utilisation prophylactique (pré traitement) de NAC augmente les taux des spermatozoïdes avec chromatine immature en comparaison de l’utilisation amélioratrice (post traitement) de NAC, et augmente les taux de fragmentation de l’ADN des spermatozoïdes par rapport aux témoins. L’utilisation de NAC en pré et post traitement augmente les taux d’oxydation par rapport aux témoins, mais augmente aussi les taux de glutathion anti-oxydant cellulaire.

**Conclusions:**

Les résultats indiquent que NAC possède la capacité de contrebalancer la toxicité induite par l’étoposide plutôt que celle d’empêcher les effets cytotoxiques de l’étoposide sur l’ADN des spermatozoïdes ; ceci suggère que l’administration de NAC aux cellules préalablement exposées à l’étoposide est préférable à son utilisation prophylactique. Comme les résultats témoignent que NAC semble être plus efficace à atténuer les effets cytotoxiques de l’étoposide sur les spermatozoïdes plutôt que d’être utilisée comme agent chimio prophylactique, ceci renforce l’idée qu’il pourrait exister un nouveau mécanisme de NAC sur l’ADN.

**Mots-clés:**

N-acétylcystéine (NAC), Etoposide, Fragmentation de l’ADN spermatique, Stress oxydatif spermatique

## Background

Testicular tumors in young men [1] are usually treated with standard combined chemotherapy, in which etoposide is widely used as a front-line adjuvant [2]. However, undesirable side effects are associated with its use [3], with later effects including decreased fertility [4, 5]. The impact of these effects on fertility potential is of particular concern to cancer patients. Spermatogenesis is affected by chemotherapeutic regimens that preferentially target the cell cycle. The BEP regimen (bleomycin, etoposide, cisplatin) often used in the treatment of testicular cancer is no exception. Briefly, bleomycin has the ability to induce DNA strand breaks [6], etoposide inhibits topoisomerase-II [7] and cisplatin is an alkylating agent that forms cross-links with DNA [8]. DNA topoisomerases are ATP-dependent nuclear enzymes that cause the DNA strand to rupture, allowing manipulation of DNA topology [1, 9]. It should be pointed out that testicular cancer has a deleterious impact on semen quality [10, 11].

In recent decades, progress in cancer treatment increased patient survival and improved quality of life. These advances included targeted therapies [12], hormone therapy [13], immunotherapy [14] and cytoprotection [6]. Tumor cells display low levels of endogenous enzymatic and non-enzymatic antioxidants and high levels of oxidative stress markers [15, 16], with elevated levels of reactive oxygen species (ROS) being shown to contribute to early events involved in onset and progression of Cancer [15]. Chemotherapy results in greater oxidative stress than that induced by cancer cells, and this inhibits cell proliferation, rendering cancer therapy less efficient [17]. Biological antioxidants are cytoprotectors as they react and scavenger ROS, thus protecting cells against lipid peroxidation and protamination, counteracting tissue injury. This led to the administration of antioxidants chemotherapeutic drugs to protect healthy cells from oxidative stress induced by antineoplastic therapy, block the side-effects of antineoplastic treatment and, by reducing ROS and peroxidation levels, also maintain responsiveness of cancer cells to chemotherapeutic drugs [15–17].

N-acetylcysteine (NAC) is a thiol compound with chemo-preventive and antioxidant properties [18]. Being a precursor of L-cysteine and reduced glutathione, it is also a free radical scavenger because it interacts with ROS [19]. Based on studies on the benefits and potential toxicity of NAC [19, 20], it has been safely used in preventing angiogenesis in vivo and endothelial cell invasion [21] as well as in attenuating the systemic immunosuppressive effects of cancer treatments [16]. In the same line of reasoning, as oxidative stress and associated mechanisms were related to male infertility [22, 23], antioxidants, including NAC, have become a highly potential instrument to protect reproductive functions in infertile men [24–26].

Despite the common use of etoposide in testicular cancer treatments, little is known about its mechanism of action and the multiplicity of side effects on male fertility. Only a few studies reported the effects of BEP chemotherapy on human sperm and spermatogenesis [27–29] and mouse meiosis and spermatogenesis [30]. There are no studies reporting the individual effects of each drug on spermatogenesis or sperm in humans, and only studies in animals dedicated to effects on spermatogenesis after exposure to etoposide [31–35] or cisplatin [36, 37].

Based on earlier research on chemotherapeutic drugs deterioration of the antioxidant defence system and antioxidant properties of NAC, the present study was designed to analyse the in-vitro effects of etoposide on human sperm motility, vitality, DNA integrity and fragmentation, and sperm oxidation-reduction (redox) potential, as well as to determine the potential of NAC to prevent the formation of ROS or to mitigate the deleterious effects of etoposide in humans.

Although etoposide is mostly used in combined chemotherapeutic regimens, the study of the isolated effects of the different chemotherapeutic agents is necessary to understand the isolated effects of each.

## Methods

### Ethics

Ethical guidelines were followed in the conduct of research, with written informed consent obtained before the beginning of the work. This work did not involve experiments on humans or animals, but only donated samples of surplus cells (fresh ejaculate spermatozoa). The approval of the Ethics Committee and the Declaration of Helsinki, revised in Tokyo 2004, on human experimentation does not apply to this work. According to the National Law on Medically Assisted Procreation (Law n° 58°/2017: (http://data.dre.pt/eli/diario/1/142/2017/0/pt/html) and the National Council on Medically Assisted Procreation guidelines (CNPMA-2015: www.cnpma.org.pt), no further authorizations were required.

### Patient selection and semen collection

Semen samples were collected by masturbation in sterile containers after a 3-day period of sexual abstinence from 20 patients who sought sperm analysis at the infertility clinic. After liquefaction, semen parameters were evaluated according to World Health Organization (WHO) guidelines [38]. For the experiments, we used only spermatozoa from ejaculated samples of normozoospermic patients enrolled in infertility treatments due to female factor. For this, we created the following inclusion criteria for men: absence of known pathologies and medication intake; normal physical examination, normal hormonal profiles and karyotypes; analysis of semen without agglutination, immature forms, leukocytes and microorganisms, a sperm volume ≥ 1.5 mL and a sperm concentration ≥ 15 × 10^6^/mL [38]. We have not had access to donated semen of fertile volunteers. The number of cases used for the present study was considered sufficient [39].

### Chemicals

Unless otherwise noted in the text, chemicals were purchased from Sigma Aldrich (St. Louis, USA).

### Experimental design

After the clinical semen analysis, the remaining ejaculate volume of each patient was centrifuged at 1500 rpm for 5 min to discard the seminal fluid and the resulting pellet was resuspended in pre-warmed sperm preparation medium (SPM; Medicult Origio, Jyllinge, Denmark) and diluted to a final concentration of 10x10^6^sperm/mL. Each sperm sample was subsequently divided into five different experimental conditions. The experiments were repeated 20 times. Each experiment had a duration of 2 h and was performed in a humidified incubator with 5% CO2 at 37 °C. The control group (CT) consisted of sperm incubated with SPM; the NAC group consisted of incubating sperm with 50 μM of NAC; the ETO (etoposide) group consisted of incubating sperm with 25 μg/mL of etoposide; the NAC-ETO group (pre-treated group) consisted of sequential incubation of sperm with 50 μM of NAC for the first hour, plus 25 μg/mL of etoposide for the second hour; and the ETO-NAC group (post-treated group) consisted of sequential incubation of sperm with 25 μg/mL of etoposide for the first hour plus 50 μM of NAC for the second hour. The dose of 25 μg/mL of etoposide was that considered pharmacologically and physiological relevant for human therapeutic doses [40] (https://pubchem.ncbi.nlm.nih.gov/compound/etoposide#section=Absorption-Distribution-and-Excretion). For NAC, the 50 μM dose was that previously determined to maximize results without damaging sperm [41]. The exposure time was based on toxicological studies that showed the highest bioavailability of the etoposide within the first 2 h (https://pubchem.ncbi.nlm.nih.gov/compound/etoposide#section=Absorption-Distribution-and-Excretion), [42].

Semen parameters were determined according to WHO 2010 guidelines [38] at the time of recovery at the in vitro fertilization (IVF) clinic. For the experimental groups, we evaluated total progressive motility, vitality, chromatin condensation, DNA fragmentation, DNA oxidative damage and glutathione levels. We did not use computer-assisted sperm analysis for sperm motility and determination of sperm motility kinematic characteristics. This methodology is presently of restricted use for research purposes that are not under the scope of the present work. Thus, the determination of sperm parameters is usually performed under WHO 2010 guidelines in IVF centers using the optical microscope. In Europe, spermiogram evaluation performed in IVF centers is evaluated periodically by ESHRE.

### Determination of sperm chromatin condensation

Chromatin condensation was evaluated by acidic aniline-blue staining. 10–20 μL of each sample were smeared on glass slides and sperm fixed with 3% glutaraldehyde in 0.2 M phosphate buffered saline (PBS, pH 7.4) for 30 min at room temperature (RT). Slides were then stained with 5% aqueous aniline-blue in 4% acetic acid (pH 3.5, 5 min, RT). After washing in PBS and air-dried, the percentage of sperm heads stained dark blue (indicates immature histone-rich nuclei) was calculated. On each slide, a minimum of 200 morphologically normal sperm were blindly evaluated on an Olympus BX41 optical microscope (Olympus Corporation, Tokyo, Japan).

### Determination of sperm DNA fragmentation

Sperm DNA fragmentation (sDNAfrag) was evaluated by the terminal deoxynucleotidyl transferase dUTP nick end labelling (TUNEL) assay using the In-Situ Cell Death Detection Kit (Roche, Mannheim, Germany) [43, 44]. 10–20 μL of each sample were smeared on glass slides and sperm fixed with 4% paraformaldehyde in PBS (1 h, RT). Slides were then washed in PBS and permeabilized with 0.1% Triton-X in 0.1% sodium citrate (2 min, 4 °C). After washing in PBS, the slides were incubated in a dark-moist chamber with 50 μL TUNEL mixture (1 h, 37 °C). Subsequently, the slides were washed in PBS and counterstained with mounting medium containing DAPI (Vectashield antifade medium containing 4′,6-diamidino-2-phenylindole, DAPI; Vector Laboratories, Burlingame, CA, USA). The number of sperm emitting green fluorescence (TUNEL-positive) was recorded as a percentage of total counted normal sperm (DAPI-stained). On each slide, a minimum of 200 morphologically normal sperm were blindly were evaluated on a Leitz DMRBE fluorescence microscope (Leica, Wetzlar, Germany). The value of the method used (TUNEL) for the evaluation of sperm DNA fragmentation has been extensively demonstrated to be of equal value to those using flow cytometry. Additionally, the flow cytometry method does not allow selective counting of sperm DNA fragmentation in morphological normal sperm [43].

### Determination of sperm DNA oxidative damage

Oxidative stress in sperm samples was measured by the detection of 8-hydroxy-2′-deoxyguanosine (8-OHdG) using the fluorescent protein binding method (OxyDNA Test) according to the manufacturer’s instructions (EKF Diagnostics, Barleben, Germany). 10–20 μL of each sample were smeared on glass slides and sperm fixed with 4% paraformaldehyde in PBS (1 h, RT). The slides were then washed (wash-solution) and permeabilized with 0.1% Triton-X in 0.1% sodium citrate (2 min, 4 °C). After washing (wash-solution), they were incubated in a dark-moist chamber with 50 μL of fluorescein isothiocyanate (FITC)-Conjugated (1 h, RT). After incubation, the slides were washed (wash-solution) and counterstained with mounting medium containing DAPI. The number of sperm emitting green fluorescence (OxyDNA-positive) was recorded as a percentage of the total counted normal sperm (DAPI-stained). On each slide, a minimum of 200 morphologically normal sperm were blindly were evaluated on a Leitz DMRBE fluorescence microscope (Leica, Wetzlar, Germany). There are several methods for the determination of sperm DNA oxidative damage. One of the predominant forms of oxidative injury induced by free radicals in DNA is 8-OHdG, which has therefore been widely used as a biomarker for oxidative stress. This biomarker has been used to estimate the DNA damage in humans following exposure to cancer-causing agents [45]. The method applied here has been demonstrated superior results [46].

### Glutathione quantification

Levels of antioxidant glutathione (GSH) were quantified for each experimental condition with the Glutathione assay kit according to the manufacturer’s guidelines (Sigma Aldrich, St. Louis, USA). Briefly, the samples were centrifuged (240 g, 10 min) and the supernatant was discarded. After washing the pellet with PBS, the samples were centrifuged (600 g, 10 min) and the supernatant discarded. Then, 20 μL of 5% 5-sulfosalicylic acid (SSA) was added, the suspension was frozen twice in liquid nitrogen and then thawed in a 37 °C bath. After centrifuging (10,000 g, 10 min), 150 μL of working-mixture was added to 10 μL of each sample. Samples were incubated 5 min and then 50 μL of NADPH (nicotinamide adenine dinucleotide phosphate) solution were added to each sample. Quantitation of glutathione was performed by sample absorbance reading on a microplate reader (BioRad Model 680, California, USA) at 415 nm, every minute over a period of 5 min.

### Statistical analysis

Statistical analysis was performed using Graph Pad Prism 7 (GraphPad Software Inc., San Diego, USA). Because of the sample size, non-parametric tests were used. The difference between the five different experimental groups was tested by the Friedman test. Post-hoc analysis between groups was conducted resorting to Dunn Test. The correlation between the different experimental groups (both for the different methods used for each group as well as within the same method among different groups) was assessed with the Spearman Rank Correlation Coefficient. Values with *P* < 0.05 were considered significant.

## Results

### Patient characteristics

The patients were of reproductive age (36.5 ± 4.2 years) and exhibited normal semen pH, viscosity and liquefaction time, without presence of round cells, leucocytes or agglutination. There were no significant differences between the patients at the time of collection for mean sperm count (127.7 ± 86.9 × 10^6^ /mL) and mean percentage of sperm total progressive motility (54.8 ± 5.2%), rapid progressive motility (33.2 ± 8.2%), normal morphology (6.6 ± 2.4%), vitality (75.8 ± 7.0%) and hypo-osmolality (73.1 ± 5.2%).

### Effects on sperm total progressive motility

Regarding the mean percentage of sperm total progressive motility, no significant differences were observed between groups (Table 1).

**Table 1:** Mean values **of studied parameters** and comparisons between groups

Parameters	TPM (%)	HOST (%)	AB+ (%)	TUNEL (%)	8-OHdG (%)
Mean values					
CT	57.0 ± 6.0	50.7 ± 5.3	21.8 ± 9.5	16.1 ± 3.2	9.3 + 4.4
NAC	68.6 ± 9.2	49.9 ± 5.2	22.2 ± 11.4	15.4 ± 2.4	23.7 ± 13.5
ETO	60.1 ± 7.1	56.4 ± 6.6	33.7 ± 11.1	23.7 ± 1.1	24 ± 16.5
NAC-ETO	49.3 ± 9.9	47.5 ± 4.8	23.2 ± 6.6	20.4 ± 4.5	26 ± 12
ETO-NAC	50.2 ± 5.2	49.4 ± 8.0	20.8 ± 9.0	19.1 ± 6.3	25.6 ± 11.7
Statistical comparisons between groups (*P* values)					
CT vs NAC	NS	NS	NS	NS	0.002
CT vs ETO	NS	NS	0.02	0.0019	0.002
CT vs NE	NS	NS	NS	0.0044	0.0421
CT vs EN	NS	NS	NS	NS	0.0283
NAC vs ETO	NS	0.04	0.04	0.02	NS
NAC vs NE	NS	NS	NS	0.0039	NS
NAC vs EN	NS	NS	NS	0.0042	NS
ETO vs NE	NS	0.01	0.0001	NS	NS
ETO vs EN	NS	0.04	0.00001	NS	NS
NE vs EN	NS	NS	0.0044	NS	NS

Values are expressed in mean ± standard deviation, TPM = sperm total progressive motility, HOST = sperm hypoosmotic swelling test, AB+ = positive sperm aniline blue staining (presence of immature chromatin), TUNEL = terminal deoxynucleotidyl transferase dUTP nick end labelling (presence of sperm DNA fragmentation), 8-OHdG = 8-hydroxy-2′-deoxyguanosine (presence of sperm oxidative damage), CT = control, NAC = N-acetylcysteine, ETO = etoposide, NAC-ETO = incubation with NAC followed by etoposide addition, ETO-NAC = incubation with etoposide followed by addition of NAC, Significant differences (*P* < 0.05), NS = not significant

### Effects on sperm membrane integrity

In relation to the mean percentage of sperm membrane integrity, group control was not significantly different to the other groups. Surprisingly, the ETO group showed significantly higher values regarding the other groups. Although not statistically significant, the NAC-ETO group (pre-treated group) presented lower values than the ETO-NAC group (post-treated group) (Table 1).

### Effects on sperm chromatin condensation

Concerning the mean percentage of sperm with immature chromatin, the ETO group exhibited higher values of sperm with uncondensed chromatin than all the other groups. The pre-treated NAC-ETO group presented a significantly higher mean percentage of sperm with immature chromatin than the post-treated ETO-NAC group (Table 1).

### Effects on sperm DNA fragmentation

Respecting the mean percentage of sDNAfrag, the control group presented no significant differences for the post-treated ETO-NAC group, while the pre-treated NAC-ETO group showed a significantly higher mean percentage of sDNAfrag than the control group. The NAC group showed significantly lower sDNAfrag levels than combined treated groups. The ETO group evidenced the highest values of sDNAfrag, but significant differences were observed only to NAC and control groups (Table 1).

### Effects on sperm oxidative profile

With reference to the mean levels of 8-OHdG, all groups evidenced significantly higher levels than the control group, with no significant differences between them. Although not significant, the NAC-ETO group exhibited higher levels of 8-OHdG than the ETO-NAC group (Table 1).

### Effects on sperm resistance to oxidative stress

**Fig. 1:**
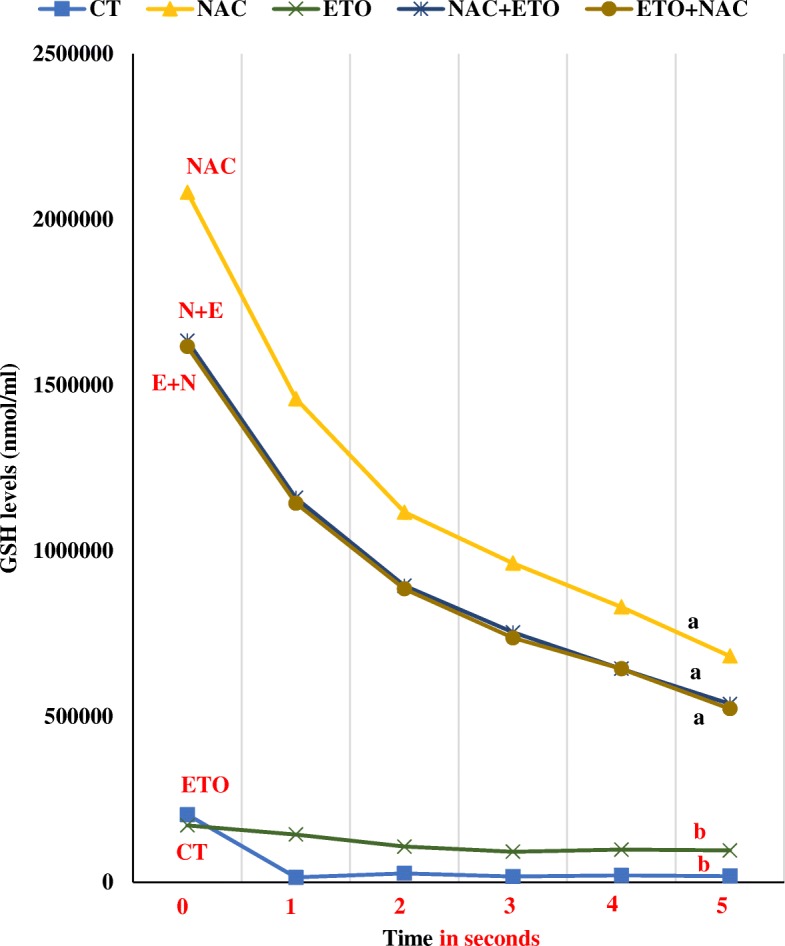
Glutathione production measured in human sperm with comparisons between groups. CT = control group (samples incubated with sperm preparation medium), NAC group = sperm treated with 50 μM of N-acetylcysteine, ETO group = sperm treated with 25 μg/mL of etoposide, NAC + ETO group = sperm pre-treated with 50 μM of NAC for the first hour plus 25 μg/mL of etoposide for the second hour of incubation, ETO + NAC group = sperm incubated with 25 μg/mL of etoposide for the first hour plus post treatment with 50 μM of NAC for the second hour. Significant differences (*P* < 0.05) between experimental groups are indicated by letters over corresponding lines (a = a, b = b, a ≠ b)

To evaluate sperm resistance to oxidative stress, antioxidant glutathione levels were measured. The control and ETO groups presented the lowest GSH levels, being significantly lower than the NAC and the combined groups, with no differences between both. The NAC group showed significantly higher levels of glutathione in relation to the control and ETO groups, but not to the combined exposed groups. Both combined exposed groups (NAC-ETO and ETO-NAC) showed significantly higher GSH levels than control and ETO groups, with no significant differences observed between them (Fig. 1).

## Discussion

Improvements in technology and research in oncology have resulted in a growing number of patients being successfully treated and surviving [47]. Though being constantly improved to obtain maximum results with minimal secondary effects, some chemotherapeutic regimens are still detrimental for male fertility [48]. Consequently, it is important to preserve fertility prior to chemotherapy. Despite efforts to encourage sperm cryopreservation before cancer treatments, many men only achieve to cryopreserve sperm after the first treatment regimen [49]. To circumvent infertility as a side-effect of oncologic treatments, it was suggested to complement treatments with cytoprotectors in order to maintain male reproductive aptitude during chemotherapy [6]. In this sense, the thiol antioxidant NAC has been reported as a possible chemoprotective agent [16].

The present results showed that, in relation to controls, sperm exposed in-vitro to the etoposide alone did not alter sperm motility and membrane integrity, but increased the rates of immature sperm chromatin, sperm DNA fragmentation and oxidative stress levels, without increasing the levels of intracellular antioxidants. In relation to the NAC group, exposure to etoposide alone evidences higher rates of immature chromatin and sDNAfrag, lower GSH rates and similar values regarding sperm oxidative stress. And in relation to the combined groups, exposure to etoposide alone evidences higher rates of immature chromatin and lower GSH rates, but similar values regarding sDNAfrag and oxidative stress. On the other hand, in relation to controls, exposure to NAC alone did not affect sperm parameters, sperm chromatin condensation and sperm DNA fragmentation. Although exposure to NAC significantly increased levels of oxidative stress than controls, it exhibited a profound increase in glutathione levels. Compared with the combined groups, exposure to NAC alone showed lower rates of sDNAfrag, with no differences regarding the other parameters evaluated. Comparisons between the combined groups showed in the NAC-ETO group a significant increase in immature chromatin, a non-significant higher mean percentage of sperm with sDNAfrag and sperm oxidative stress, a non-significant lower rate of sperm with membrane integrity and a similar rate of GSH levels.

In the current study, we evaluated the capacities of NAC as a prophylactic (pretreatment) or ameliorator (post-treatment) agent over the effects caused on sperm during in-vitro exposure to etoposide, a chemotherapeutic drug frequently used in testicular cancer treatment. For this purpose, human sperm samples were incubated with etoposide and pre- or post-treated with NAC. The results revealed that the prophylactic use of NAC (NAC-ETO: exposure of sperm to NAC followed by exposure to etoposide) increased the rates of immature sperm chromatin in relation to ameliorator use of NAC (ETO-NAC: exposure of sperm to etoposide followed by exposure to NAC), and increased the rates of sperm DNA fragmentation compared to controls.

As NAC-ETO vs ETO-NAC were significantly different regarding sperm chromatin immaturity (NAC-ETO had higher immature sperm chromatin) and not -significantly different relative to sDNAfrag (NAC-ETO had higher sDNAfrag); that NAC-ETO and ETO-NAC did not differ from controls for sperm chromatin immaturity and only NAC-ETO significantly differed from controls regarding sDNAfrag (NAC-ETO had higher sDNAfrag); that sperm chromatin immaturity in NAC was similar to NAC-ETO and ETO-NAC, and that sDNAfrag in NAC had lower sDNAfrag then NAC-ETO (higher) and ETO-NAC (less higher); and that in relation to the ETO, NAC-ETO and ETO-NAC groups exhibited lower (significant) sperm chromatin immaturity, lower (non-significant) sDNAfrag, and higher (significant) GSH levels, it is evident that NAC exerts a beneficial effect over etoposide exposure. Notwithstanding, in relation to ETO, the NAC-ETO and ETO-NAC groups exhibited lower (significant) membrane integrity and greater (non-significant) sperm oxidative stress. Results thus suggest that the prophylactic use of NAC seems to have no better beneficial effects than the addition of NAC to cells previously exposed to etoposide.

Previous studies in patients treated with BEP revealed a reduction in testicular function and sperm quality, with results showing: a decrease in sperm concentration, progressive motility and normal morphology [27]; an increase in sperm aneuploidies [28, 29]; and an increase in serum FSH levels, sperm DNA decondensation, DNA fragmentation and aneuploidies, in association with a decrease in sperm concentration [29]. However, these effects are the result of a combination of bleomycin, etoposide and cisplatin. Regarding etoposide alone, there are no studies in humans.

Numerous beneficial effects against early stages of toxicity-induced damages have been attributed to NAC [50]. In this context, NAC has been shown to induce apoptosis in several transformed cell lines but not in normal cells [51], suggesting that antioxidants have stronger effects on cells already under stress [22, 23]. In sperm, it has been suggested that NAC is not likely to neutralize the effects of etoposide on sperm DNA by a DNA repair mechanism, since post-ejaculated sperm lack DNA repair systems [16]. Similarly, in other cell types, it has been reported that NAC may have a pro-oxidant effect [52, 53], and pro-oxidant environments have been associated with a decrease in sperm quality [22, 23].

Antioxidants also reduce oxidative free radicals created by chemotherapeutic drugs [15, 16], and their use in fertility preservation could be beneficial in cancer treatments once gonadal toxicity is mainly induced through oxidative stress injury [54]. This is of particular importance since sperm are highly sensitive to ROS because they possess a limited number of cytoplasmic antioxidants [55] and about 25–40% of infertile men feature high levels of ROS, which results in deleterious lipid peroxidation and protein oxidation [22, 23]. In the present report, we evaluated the protective role of NAC in relation to oxidative stress imposed on sperm from exposure to etoposide. By quantification of 8-OHdG levels, we observed a significant increase in oxidative stress in all groups regarding controls, with no differences between the other groups. In these experiments, only a slight non-significant lower oxidative level was observed in etoposide-exposed sperm treated with NAC as an ameliorator agent (ETO-NAC). This observed difference in oxidative levels could, to a certain extent, explain the slightly better results obtained for motility and viability, as ROS may suppress sperm function [22, 23].

The antioxidant activity of NAC has not only been attributed to a fast reaction with free radicals, but also to the restitution of reduced glutathione (GSH). Current results revealed significantly higher sperm intracellular GSH levels after incubation with NAC. However, because similar values were obtained at combined exposures, it was not possible to discern which combination is best to reduce etoposide toxicity. As far as we are aware, this is the first report showing that sperm have the ability to take up exogenous glutathione.

## Conclusion

Our results evidenced that NAC seems to be more efficient in attenuating sperm etoposide cytotoxic effects instead of being used as a chemoprophylactic agent, which reinforces the idea that there might be a new NAC mechanism over DNA. The results also clearly indicated that NAC induces a profound increase in GSH levels, which confirms its antioxidant properties. However, since NAC sequentially combined with etoposide also showed significant increases in sDNAfrag and 8-OHdG levels, it will be necessary in the future to test the effects of NAC combined with other powerful antioxidants and to assess their combined ability to preserve sperm against etoposide. Accordingly, our unpublished data indicates that the best approach would be the simultaneous use of etoposide and NAC. Additionally, as this study focused on etoposide, there are no certainties on NAC competence against other types of chemotherapeutic drugs and thus it will be necessary to assess its cytoprotector abilities against other agents, especially since in the clinical setting the combined therapies are mostly used. As the present results indicate that NAC may be more able to counteract the etoposide-induced toxicity than to prevent the etoposide cytotoxic effects over sperm DNA, it is possible to suggest that administration of NAC to cells formerly exposed to the etoposide is preferable to its prophylactic use. However, this should be confirmed using a higher number of subjects before applying a future decision in clinical studies for which the present study was not designed.

## Abbreviations

BEP: Bleomycin + etoposide + cisplatin
CT: Control group
DAPI: 4′,6-diamidino-2-phenylindole
ETO: Etoposide
FITC: Fluorescein isothiocyanate
GSH: Antioxidant glutathione
IVF: In vitro fertilization
NAC: N-acetylcysteine
NADPH: Nicotinamide adenine dinucleotide phosphate
8-OHdG: 8-hydroxy-2’-deoxyguanosine
PBS: Phosphate buffered saline
ROS: Reactive oxygen species
RT: Room temperature
sDNAfrag: Sperm DNA fragmentation
SSA: 5-sulfosalicylic acid
SPM: Sperm preparation medium
TUNEL: Terminal deoxynucleotidyl transferase dUTP nick end labelling assay
WHO: World Health Organization

## References

[1] Albers P, Albrecht W, Algaba F, Bokemeyer C, Cohn-Cedemark G, Fizazi K (2015). Guidelines on testicular cancer: 2015 update. Eur Urol.

[2] Calabrò F, Albers P, Bokemeyer C, Martin C, Einhorn LH, Horwich A (2012). The contemporary role of chemotherapy for advanced testis cancer: a systematic review of the literature. Eur Urol.

[3] Fung C, Fossa SD, Williams A, Travis LB (2015). Long-term morbidity of testicular cancer treatment. Urol Clin North Am.

[4] Cvancarova M, Samuelsen SO, Magelssen H, Fossa SD (2009). Reproduction rates after cancer treatment: experience from the Norwegian radium hospital. J Clin Oncol.

[5] Williams DH, Karpman E, Sander JC, Spiess PE, Pisters LL, Lipshultz LI (2009). Pretreatment semen parameters in men with cancer. J Urol.

[6] Rabaça A, Sousa M, Alves M, Oliveira PF, Sá R (2015). Novel drug therapies for fertility preservation in men undergoing chemotherapy: clinical relevance of protector agents. Curr Med Chem.

[7] Chen AY, Liu LF (1994). DNA topoisomerases: essential enzymes and lethal targets. Ann Rev Pharmacol Toxicol.

[8] Kelland L (2007). The resurgence of platinum-based cancer chemotherapy. Nat Rev Cancer.

[9] Froelich-Ammon SJ, Osheroff N (1995). Topoisomerase poisons: harnessing the dark side of enzyme mechanism. J Biol Chem.

[10] Auger J, Sermondade N, Eustache F. Semen quality of 4480 young cancer and systematic disease patients: baseline data and clinical considerations. Basic Clin Androl. 2016;26(3). 10.1186/s12610-016-0031-x. 10.1186/s12610-016-0031-x PMC4758099 26893905

[11] Caponecchia L, Cimino G, Sacchetto R, Fiori C, Sebastianelli A, Salacone P (2016). Do malignant diseases affetc semen quality? Sperm parameters of men with cancer. Andrologia.

[12] Santo L, Siu KT, Raje N (2015). Targeting cyclin-dependent kinases and cell cycle progression in human cancers. Semin Oncol.

[13] Reznikov A (2015). Hormonal impact on tumor growth and progression. Exp Oncol.

[14] Michot JM, Bigenwald C, Champiat S, Collins M, Carbonnel F, Postel-Vinay S (2016). Immune-related adverse events with immune checkpoint blockade: a comprehensive review. Eur J Cancer.

[15] Fuchs-Tarlocsky V (2013). Role of antioxidants in cancer therapy. Nutrition.

[16] Thyagarajan A, Sahu RP (2018). Potential contributions of antioxidants to cancer therapy: immunomodulation and radiosensitization. Integr Cancer Ther.

[17] Drisko JA, Chapman J, Hunter VJ (2003). The use of antioxidant therapies during chemotherapy. Gynecol Oncol.

[18] Aruoma OI, Halliwell B, Hoey BM, Butler J (1989). The antioxidant action of N-acetylcysteine: its reaction with hydrogen peroxide, hydroxyl radical, superoxide, and hypochlorous acid. Free Rad Biol Med.

[19] Bonanomi L, Gazzaniga A (1980). Toxicological, pharmacokinetic and metabolic studies on acetylcysteine. Eur J Respir Dis Suppl.

[20] Johnston RE, Hawkins HC, Weikel JH (1983). The toxicity of N-acetylcysteine in laboratory animals. Semin Oncol.

[21] Cai T, Fassina G, Morini M, Aluigi MG, Masiello L, Fontanini G (1999). N-acetylcysteine inhibits endothelial cell invasion and angiogenesis. Lab Investig.

[22] Agarwal A, Virk G, Ong C, du Plessis SS (2014). Effect of oxidative stress on male reproduction. World J mens Health.

[23] Bisht S, Faiq M, Tolahunase M, Dada R (2017). Oxidative stress and male infertility. Nat Rev Urol.

[24] Agarwal A, Nallella KP, Allamaneni SSR, Said TM (2004). Role of antioxidants in treatment of male infertility: an overview of the literature. Reprod BioMed Online.

[25] Ciftci H, Verit A, Savas M, Yeni E, Erel O (2009). Effects of N-acetylcysteine on semen parameters and oxidative/antioxidant status. Urology.

[26] Kefer JC, Agarwal A, Sabanegh E (2009). Role of antioxidants in the treatment of male infertility. Int J Urol.

[27] Stephenson W, Poirier SM, Rubin L, Einhorn LH (1995). Evaluation of reproductive capacity in germ cell tumor patients following treatment with cisplatin, etoposide, and bleomycin. J Clin Oncol.

[28] De Mas P, Daudin M, Vincent MC, Bourrouillou G, Calvas P, Mieusset R (2001). Increased aneuploidy in spermatozoa from testicular tumour patients after chemotherapy with cisplatin, etoposide and bleomycin. Hum Reprod.

[29] Ghezzi M, Berretta M, Bottacin A, Palego P, Sartini B, Cosci I (2016). Impact of Bep or carboplatin chemotherapy on testicular function and sperm nucleus of subjects with testicular germ cell tumor. Front Pharmacol.

[30] Bagheri-Sereshki N, Hales BF, Robaire B (2016). The effects of chemotherapeutic agents, bleomycin, etoposide, and cisplatin, on chromatin remodeling in male rat germ cells. Biol Reprod.

[31] Russell LB, Hunsicker PR, Johnson DK, Shelby MD (1998). Unlike other chemicals, etoposide (a topoisomerase-II inhibitor) produces peak mutagenicity in primary spermatocytes of the mouse. Mutat Res.

[32] Russell LB, Hunsicker PR, Hack AM, Ashley T (2000). Effect of the topoisomerase-II inhibitor etoposide on meiotic recombination in male mice. Mutat Res.

[33] Matulis S, Handel MA (2006). Spermatocyte responses in vitro to induced DNA damage. Mol Reprod Dev.

[34] Marchetti F, Pearson FS, Bishop JB, Wyrobek AJ (2006). Etoposide induces chromosomal abnormalities in mouse spermatocytes and stem cell spermatogonia. Hum Reprod.

[35] Okada FK, Stumpp T, Miraglia SM (2009). Carnitine reduces testicular damage in rats treated with etoposide in the prepubertal phase. Cell Tiss Res.

[36] Reddy KP, Madhu P, Reddy PS (2016). Protective effects of resveratrol against cisplatin-induced testicular and epididymal toxicity in rats. Food Chem Toxicol.

[37] Afsar T, Razak S, Khan MR, Almajwal A (2017). Acacia hydaspica ethyl acetate extract protects against cisplatin-induced DNA damage, oxidative stress and testicular injuries in adult male rats. BMC Cancer.

[38] World Health Organization (WHO) (2010). WHO laboratory manual for the examination and processing of human semen.

[39] Charan J, Kantharia ND (2013). How to calculate sample size in animal studies?. J Pharmacol Pharmacother.

[40] Joel S (1996). The clinical pharmacology of etoposide: an update. Cancer Treat Rev.

[41] Dorato MA, Engelhardt JA (2005). The no-observed-adverse-effect-level in drug safety evaluations: use, issues, and definition(s). Reg Toxicol Pharmacol.

[42] Slevin ML (1991). The clinical pharmacology of etoposide. Cancer.

[43] Bucar S, Goncalves A, Rocha E, Barros A, Sousa M, Sá R (2015). DNA fragmentation in human sperm after magnetic-activated cell sorting. J Assist Reprod Genet.

[44] Sá R, Cunha M, Rocha E, Barros A, Sousa M (2015). Sperm DNA fragmentation is related to sperm morphological staining patterns. Reprod BioMedicine Online.

[45] Valavanidis A, Vlachogianni T, Fiotakis C (2009). 8-hydroxy-2′-deoxyguanosine (8-OHdG): a critical biomarker of oxidative stress and carcinogenesis. J Environ Sci Health, Part C.

[46] Vorilhon S, Brugnon F, Kocer A, DEollet S, Bourgne C, Berger M (2018). Accuracy of human sperm DNA oxidation quantification and threshold determination using an 8-OHdG immuno-detection assay. Hum Reprod.

[47] Siegel R, DeSantis C, Virgo K, Stein K, Mariotto A, Smith T (2012). Cancer treatment and survivorship statistics, 2012. CA Cancer J Clin.

[48] Magelssen H, Brydoy M, Fossa SD (2006). The effects of cancer and cancer treatments on male reproductive function. Nat Clin Pract Urol.

[49] Tournaye H, Dohle GR, Barratt CLR (2014). Fertility preservation in men with cancer. Lancet.

[50] Dhouib IE, Jallouli M, Annabi A, Gharbi N, Elfazaa S, Lasram MM (2016). A minireview on N-acetylcysteine: an old drug with new approaches. Life Sci.

[51] Liu M, Pelling JC, Ju J, Chu E, Brash DE (1998). Antioxidant action via p53-mediated apoptosis. Cancer Res.

[52] Lohrke B, Xu J, Weitzel JM, Krüger B, Goldammer T, Viergutz T (2010). N-acetylcysteine impairs survival of luteal cells through mitochondrial dysfunction. Cytometry Part A.

[53] Sagara J, Bannai S, Shikano N, Makino N (2010). Conflicting effects of N-acetylcysteine on purified neurons derived from rat cortical culture. Neuroreport.

[54] Meistrich ML (2009). Male gonadal toxicity. Pediatr Blood Cancer.

[55] Zini A, Gabriel MS, Baazeem A (2009). Antioxidants and sperm DNA damage: a clinical perpective. J Assist Reprod Genet.

